# Evaluation of the antibacterial potential of silver nanoparticles synthesized through the interaction of antibiotic and aqueous callus extract of *Fagonia indica*

**DOI:** 10.1186/s13568-019-0797-2

**Published:** 2019-05-27

**Authors:** Muhammad Adil, Tariq Khan, Muhammad Aasim, Ayaz Ali Khan, Muhammad Ashraf

**Affiliations:** 1 Department of Biotechnology, University of Malakand, Chakdara Dir Lower, Pakistan; 2 Department of Chemistry, Quaid-i-Azam University Islamabad, Islamabad, 45320 Pakistan

**Keywords:** AgNPs, *F. indica*, Callus extract, Ciprofloxacin, Antibacterial activity

## Abstract

*Fagonia indica* is a widely known medicinal plant. The extracts of *Fagonia* species contain secondary metabolites such as flavonoids, phenolic compounds, and terpenoids. Silver nanoparticles are known for antibacterial properties. In this study, AgNPs were synthesized using the callus extract of *F. indica* as a reducing agent. Characterization through different techniques suggests that the AgNPs absorbed light and gave SPR peaks at 414 nm while in case of ciprofloxacin supplemented callus mediated AgNPs the peak were recorded at 419 nm. Furthermore, FTIR analysis revealed the role of amides, acyl group, nitro group of callus extract of *F. indica*, and some functional groups of the ciprofloxacin in the reduction process as well as the capping and stabilization of AgNPs. Similarly, X-Ray Diffraction analysis indicate the structure of AgNPs as face-centered cubic crystalline particles. The antibacterial activity of AgNPs and ciprofloxacin and callus extract as control against resistant bacteria such as *Escherichia coli, Citrobacter amalonaticus*,* Shigella sonnei*, and *Salmonella typhi* was studied. The combination of AgNPs and antibiotic showed better antibacterial activity as compared to AgNPs alone and ciprofloxacin alone. Maximum inhibition zone of *E. coli*,* C. amalonaticus*,* S. sonnei*, and *S. typhi* in response to AgNPs and ciprofloxacin was 38.5 mm, 35.5 mm, 33 mm, and 35.5 mm, respectively. It can, therefore, be suggested that the AgNPs along with Ciprofloxacin might have worked in interaction and resulted in better antibacterial activity against all the tested pathogens.

## Introduction

Nanotechnology focuses on manufacturing nanotechnology-based particles i.e. nanoparticles (Vidhu et al. 2011). Nanotechnology has gained much importance in areas such as biomedical sciences, chemical industry, drug-gene delivery, and energy sciences (Iravani 2011). The demand for production of metal nanoparticles has widely increased during the recent past due to their use in different areas such as chemical sensors, biosensing, antimicrobial activity, energy, medicine, and catalysis (Edison and Sethuraman 2012; Iravani 2011). In the recent years, metal nanoparticles attracted the researchers due to their applications in biological tagging and pharmaceutics (Begum et al. 2009). Size, shape and surface morphology of nanoparticles defines its physical and chemical properties (Philip 2010). Furthermore, the interactions of metal salts with the stabilizer, the media present in the surrounding as well as the way adopted for the synthesis affects the properties of nanoparticles. Thus, controlled synthesis of nanoparticles is key to attain better-applied characteristics of these nanoparticles. Among the metal nanoparticles, the AgNPs have been widely studied due to their applications in many sectors of life (Le et al. 2010). AgNPs have gained the state of a significant class of nanoparticles. AgNPs are now mainly used as an antifungal and antibacterial agent (Rauwel et al. 2015). The dimensions of the nanoparticle influence the antimicrobial activity of AgNPs. Smaller the dimensions of the particles, greater will be the antimicrobial effect of the particles. AgNPs as an antimicrobial agent can be utilized for the cleaning and decontamination of wastewater treatment plants, for the prevention of bacterial colonies and elimination of microorganisms to protect food and textile fabrics (Guzman et al. 2012).

However, the broad and abusive use of these agents has led to bacterial resistance to antibiotics (Hajipour et al. 2012). Various strains of bacteria are developing resistance to much of the currently available antibiotics and posing a serious threat to the public health (Lynch et al. 2007). Different studies have reported that drug-resistant infections are increasing day by day (Boucher et al. 2009; Dasari et al. 2014). Antibiotic resistance affects both intrahospital pathogens as well as nonhospital strains. It has increased both morbidity and mortality rate thus became a global threat for human life (Franci et al. 2015). Antibiotics resistant strains emerged due to the continuous use of topical and systemic antimicrobial agents (Cooper et al. 2002). Antibiotics fail to effectively treat the infections initiated by the drug-resistant bacteria. Such antibiotics resistance demands for a better alternative solution (Taylor et al. 2002). Consequently, there is a higher need for the development of new bactericides to avoid a potential threat to public health. This attracts the focus of researchers to the field of nanotechnology (Morones et al. 2005).

Metal nanoparticles are synthesized via different routes (Satyavani et al. 2011). They can be synthesized both chemically and biologically. Chemical methods are too costly and it involves the use of toxic capping and reducing agents such as sodium borohydride, hydrazine, and dimethylformamide (Hebeish et al. 2010). In addition, nanoparticles synthesized through chemical method may lead to adverse effects in biomedical applications (Shankar et al. 2004). Thus, there is an increasing need to use eco-friendly methods that are cost-effective, non-toxic and have a high yield (Dipankar and Murugan 2012). Biosynthesis of nanoparticles make use of biogenic extracts for example plants extract (Satyavani et al. 2011) for the production of nanoparticles on large scale (Aromal and Philip 2012). Plant-based synthesis is gaining importance due to its economical, cost-effective and eco-friendly nature. Among different herbs and shrubs, *Fagonia indica* has attracted the focus of researchers as a potent medicinal plant with an interesting profile of medicinal compounds that can be employed for the bio-reduction of silver salts to nanoparticles.

In this study, we used the callus extract of *F. indica* for the synthesis of AgNPs. The purpose of the study was to synthesize the nanoparticles in the presence of combination of an antibiotic and callus extract from the *F. indica* to assess the effects of antibiotic supplementation on AgNPs synthesis. This study also aims to compare the effects of AgNPs synthesized via callus extract alone and callus extract + antibiotic combination based AgNPs on the pathogenic bacterial strains.

## Materials and methods

### Preparation of callus extract

Callus cultures of *F. indica* were obtained through a protocol described by Khan et al. (2016). 2 g of powdered callus was added to 100 mL of distilled water and heated at high temperature i.e. up to 300 °C to obtain the callus extract. The solution was boiled for 10 min and was then allowed to cool down. It was then filtered through Whatman 2.5 µm filter paper and stored for use in the synthesis of nanoparticles.

### Synthesis of silver nanoparticles

Silver nitrate solution (4 mM) was prepared and used for the synthesis of AgNPs. The protocol was optimized at 1:1. This means that an equal volume of AgNO_3_ solution and callus extract of *F. indica* was added to the reaction tube. The reaction tubes were kept in a falcon tube rack in a shaded area illuminated by midday sunlight for 3 h. The intensity of the daylight was recorded around 20,000 lux on average. The dark brownish color is an indication of the synthesis of AgNPs. In addition, mixture of ciprofloxacin and callus extract of *F. indica* was also used for the reduction of AgNO_3_ to AgNPs. A stock solution of ciprofloxacin 0.3 mg/mL (300 µg/mL) was prepared for use in combination with AgNPs. The ratio of AgNO_3_ to callus extract to ciprofloxacin was optimized at 1:1:0.5. This means that 20 mL of 4 mM AgNO_3_ solution was mixed with 20 mL of callus extract of *F. indica*. Then, 10 mL ciprofloxacin solution was taken from the diluted stock and added to one of the AgNO_3_—Extract reactions. The tube was kept in sunlight for 3 h. Both types of reaction tubes were subject to sonication at 20 °C for 30 min before analysis by UV–Vis spectroscopy. Finally, the reaction mixtures were washed five times through ultracentrifugation at 13,000 rpm for 10 min. The resulted pellet was dried and used for further analysis.

### Characterization of AgNPs

#### UV–visible spectroscopy

To confirm the synthesis of AgNPs, the samples were analyzed using UV–Vis spectroscopy on UV-1280 Shimadzu spectrophotometer. For all the three reactions (Callus based AgNPs, Callus + Ciprofloxacin based AgNPs and Control), the UV–Vis spectrum was recorded between 350 and 650 nm. Before UV–Vis analysis, all the solutions were diluted twofold in equal volume of distilled water. The final 2 mL of the AgNPs was subject to the final UV–Vis analysis. The reaction process between silver ions and callus extract was monitored through UV–vis spectra of nanoparticles in aqueous solution.

#### Fourier transform infrared spectroscopy

Fourier transform infrared spectroscopy (FTIR) was performed to determine the possible functional groups involved in the synthesis of AgNPs. First, 2 mL AgNPs solution was taken from each reaction and added to a separate Eppendorf tube. After that, centrifugation was performed at 13,000 rpm for 10 min using minicentrifuge to wash the AgNPs with distilled water. The temperature was kept at 9 °C. This step was performed five times to properly wash the AgNPs. The AgNPs sample in liquid form was then used for FTIR analysis. The Perkin-Elmer model spectrometer was used for FTIR analysis. The transmission mode with potassium bromide (KBr) pellets in the range of 3500–500 cm^−1^ at room temperature was used during the analysis. Spectra of callus extract mediated AgNPs, Callus + Ciprofloxacin based AgNPs and only Ciprofloxacin solution were recorded to identify the functional groups involved in the AgNPs synthesis and ultimately stabilization.

#### X-Ray diffraction analysis

X-Ray diffraction (XRD) was performed to study the crystalline nature of AgNPs. For XRD analysis, dried form of the biogenic AgNPs were placed in the Shimadzu-Model, XRD 6000. The scanning mode at 40 kV with a current of 30 mA and Cu/Ka radiation (in the 2q range of 20–80) was used for recording the diffraction pattern. The average particle size of the synthesized AgNPs was determined using the Debye–Scherrer equation.

$${\text{D }} = {\upkappa} {\uplambda} /{\upbeta} {\text{ cos}}\uptheta$$

where D represents crystal size, κ represents shape factor (the ideal value of κ is 0.94), λ represents the X-ray wavelength (λ = 1.5418 A°), β represents the full width in radians at half maximum, and θ gives the Bragg's angle.

#### Antibacterial activity of AgNPs

The antibacterial activity of AgNPs was carried out against four pathogenic strains *Escherichia coli* (ATCC 23716), *Citrobacter amalonaticus* (ATCC 25405)*, Shigella sonnei* (ATCC 29930)*,* and *Salmonella typhi* (ATCC 35664). The disk diffusion method was performed as the antibacterial assay. The pure cultures of all the tested pathogens were inoculated to fresh nutrient broth in separate tubes. The tubes were incubated at 37 °C for 24 h. After 24 h, turbidity was adjusted to 1 × 10^8^ CFU/mL at a wavelength of 600 nm using a spectrophotometer. The surface of the agar plates was then fully swabbed with different bacterial strains completely ensuring that all the plates receive enough bacterial colonies. The cellulose paper discs were placed in their respective positions on the plates using sterile forceps. The plates for each bacterial strain was used in triplicate. Different volumes (5 µL, 10 µL, 15 µL, 20 µL) of callus extract mediated AgNPs, ciprofloxacin + callus extract mediated AgNPs, were added to sterile discs such that each plate receive all the concentrations of the AgNPs. Callus extract-only and ciprofloxacin-only discs were used as a control for the activity. The plates were incubated overnight at 37 °C. The zones of inhibitions were then observed and measured on the next day using Vernier caliper.

## Results

### Characterization of AgNPs

#### UV–visible spectra analysis

**Fig. 1:**
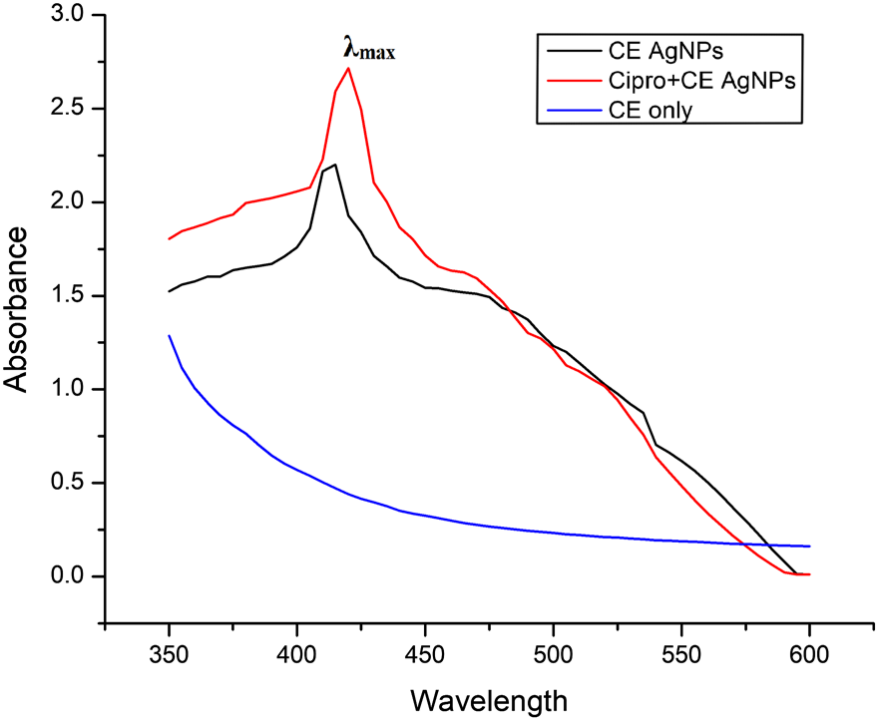
UV–Vis spectra of callus extract (CE) mediated AgNPs and ciprofloxacin + callus extract mediated AgNPs compared with control (callus extract only)

AgNPs were synthesized through reduction of AgNO_3_ in various reaction mixtures. AgNPs absorbed the radiation in a range between 350 and 650 nm. UV–Vis spectroscopy was performed for all the three reactions. In the case of callus extract mediated AgNPs, the maximum absorbance (λ_max_) was recorded at 414 nm while in the case of ciprofloxacin + callus extract mediated AgNPs, the absorption maxima (λ_max_) was recorded at 419 nm (Fig. 1). It is generally observed that the λ_max_ is between 410 and 440 nm for the AgNPs to have a desired size. However, there is a shift in the surface plasmon resonance peaks toward higher wavelengths in the case of ciprofloxacin + callus extract mediated AgNPs. The reason for this shift has been suggested to be the involvement of ciprofloxacin in the reduction process for the biosynthesis of AgNPs (Shah et al. 2014).

#### Fourier transform infrared spectroscopy

**Fig. 2:**
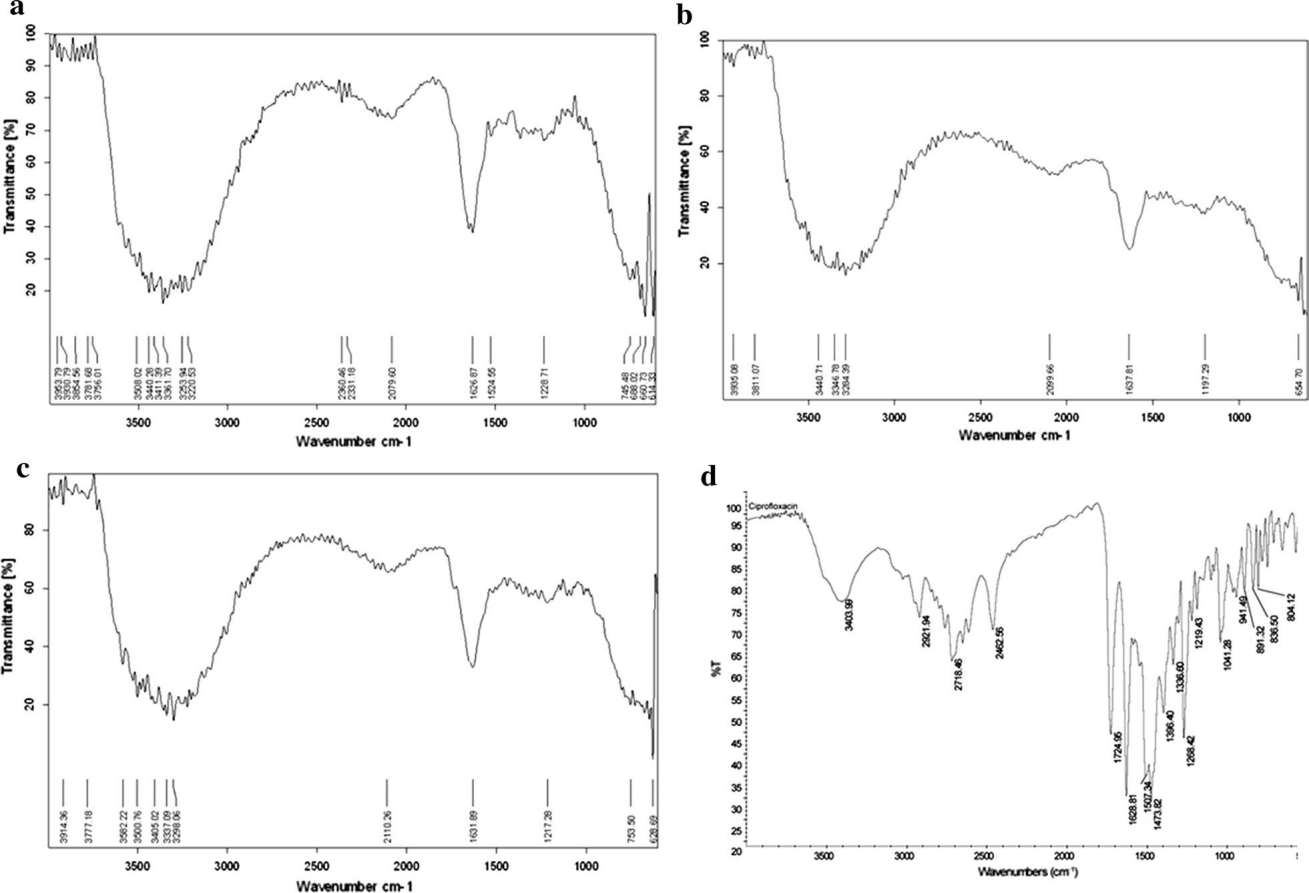
FTIR spectra of** a** callus extract of *F. indica*,** b** callus extract mediated AgNPs,** c** ciprofloxacin + callus extract mediated AgNPs and **d** ciprofloxacin as control

FTIR spectroscopy was performed for the qualitative analysis of the expected reducing and capping compounds/metabolites involved in the synthesis of AgNPs. In this study, the FTIR spectra of callus extract of *F. indica* showed major peaks at 3508 cm^−1^, 3440 cm^−1^, 3411 cm^−1^, 3361 cm^−1^, 3253 cm^−1^, 3220 cm^−1^, 1626 cm^−1^, 1524 cm^−1^ and 1228 cm^−1^ (Fig. 2a). The peaks from 3508 to 3220 cm^−1^ corresponds to the bending vibrations of amide II of proteins while the peak at 1626 cm^−1^ corresponds to the N–H stretching vibrations of amide I of proteins present in the callus extract of *F. indica*. The peaks at 3361 cm^−1^, 3253 cm^−1^, 3220 cm^−1^ also corresponds to the O–H stretching vibrations of alcohols and phenols. The peak at 1524 cm^−1^ corresponds to a nitro group of a compound while the peak at 1228 cm^−1^ corresponds to the acyl group (Fig. 2).

#### X-Ray diffraction analysis of AgNPs

**Fig. 3:**
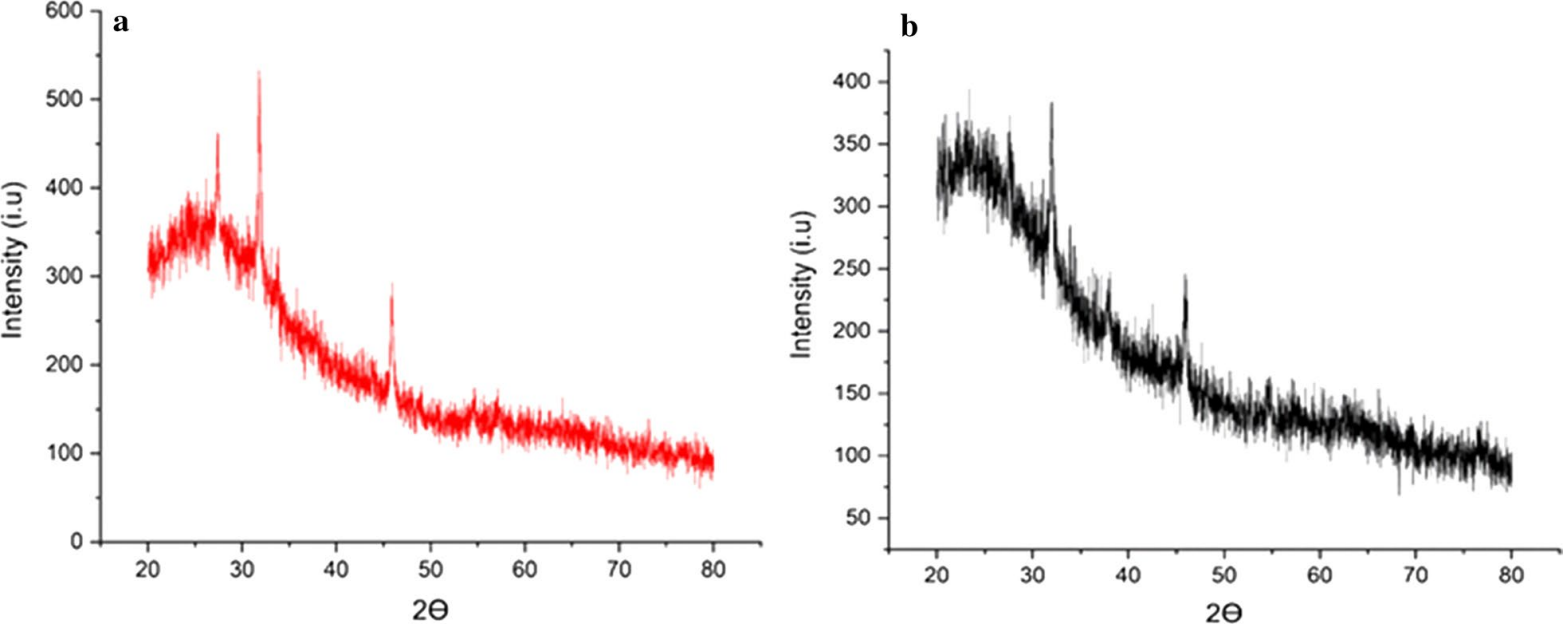
XRD spectra of** a** callus extract mediated AgNPs and** b** ciprofloxacin + callus extract mediated AgNPs

XRD analysis was performed to study the crystalline nature of AgNPs. The distinctive diffraction peaks at 2 theta values of 27.2°, 32° and 46.53° can be indexed to the (111), (220) and (311) reflection planes of the face-centered cubic structure of silver (Fig. 3a). In case of ciprofloxacin + callus extract mediated AgNPs, the X-Ray diffractogram showed distinct peaks at 2 theta values of 27.3°, 31.2° and 46.2° can be indexed to the (111), (220) and (311) reflection planes of the face-centered cubic structure of silver (Fig. 3b). However, these peaks are not as sharp as the one in Fig. 3a.

## Antibacterial activity of AgNPs

**Fig. 4:**
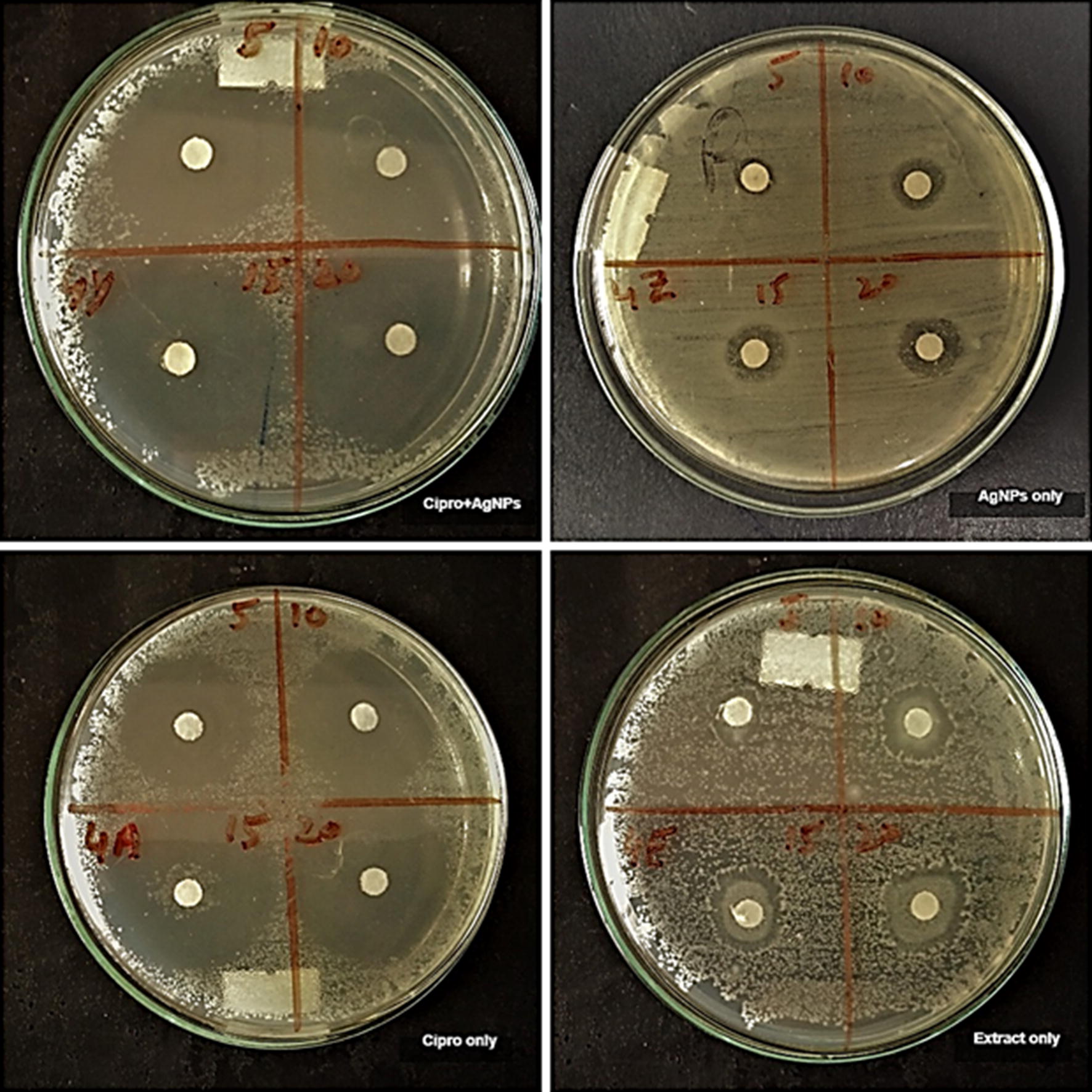
Activity of ciprofloxacin + callus extract mediated AgNPs, callus extract mediated AgNPs, ciprofloxacin only and extract only controls against Escherichia coli

**Fig. 5:**
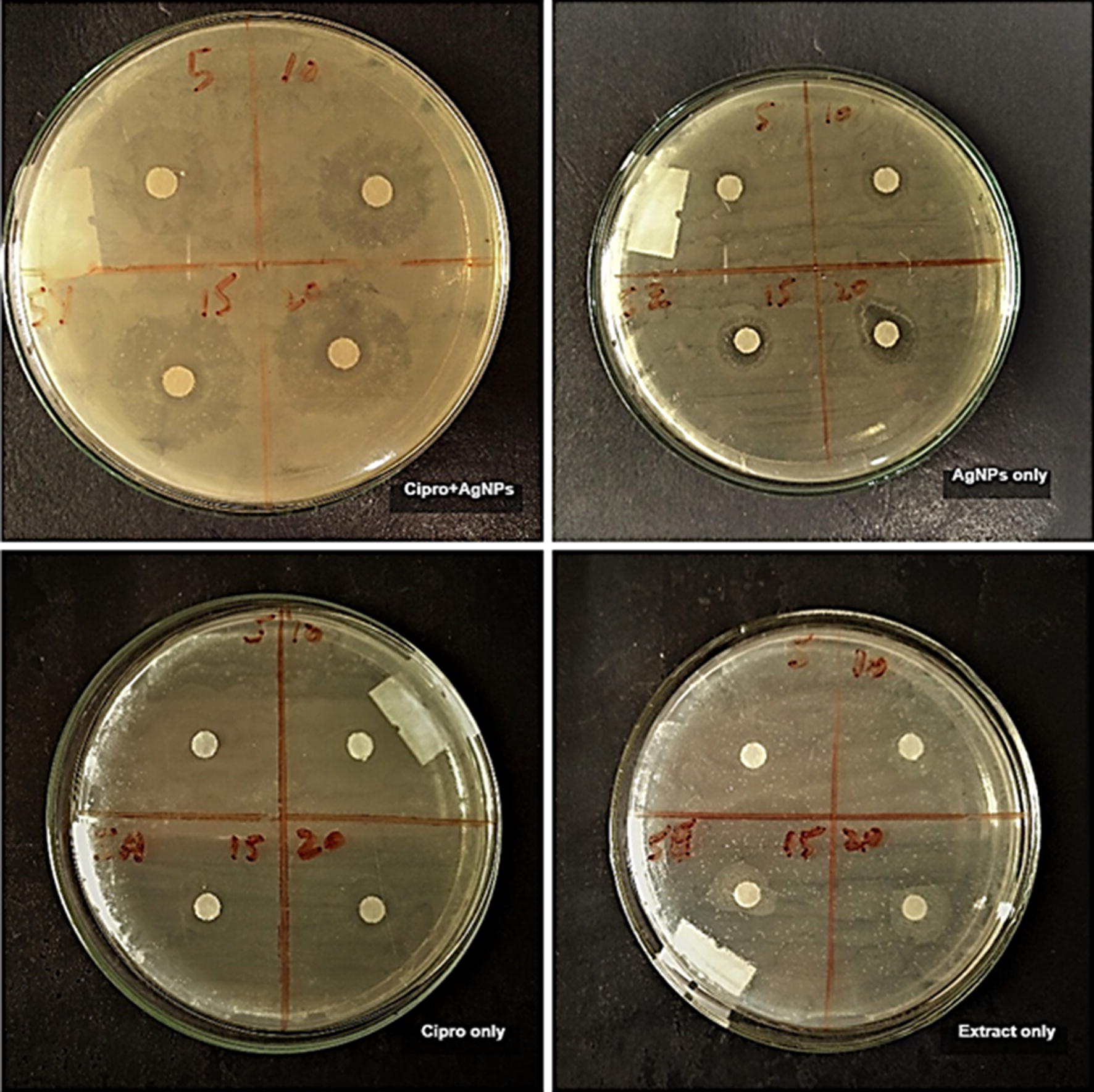
Activity of ciprofloxacin + callus extract mediated AgNPs, callus extract mediated AgNPs, ciprofloxacin only and extract only controls against Citrobacter amalonaticus

**Fig. 6:**
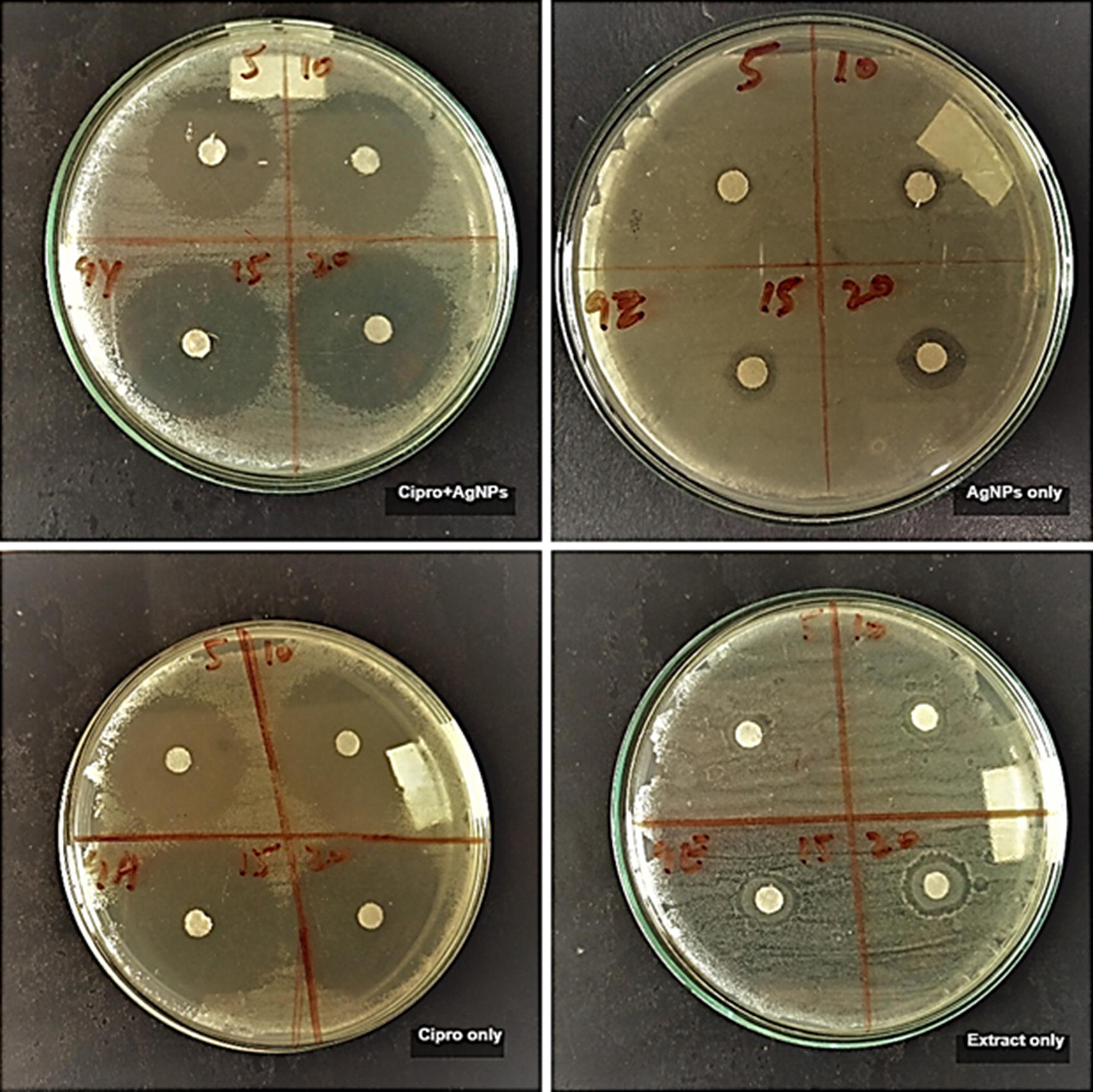
Activity of ciprofloxacin + callus extract mediated AgNPs, callus extract mediated AgNPs, ciprofloxacin only and extract only controls against Shigella sonnei

**Fig. 7:**
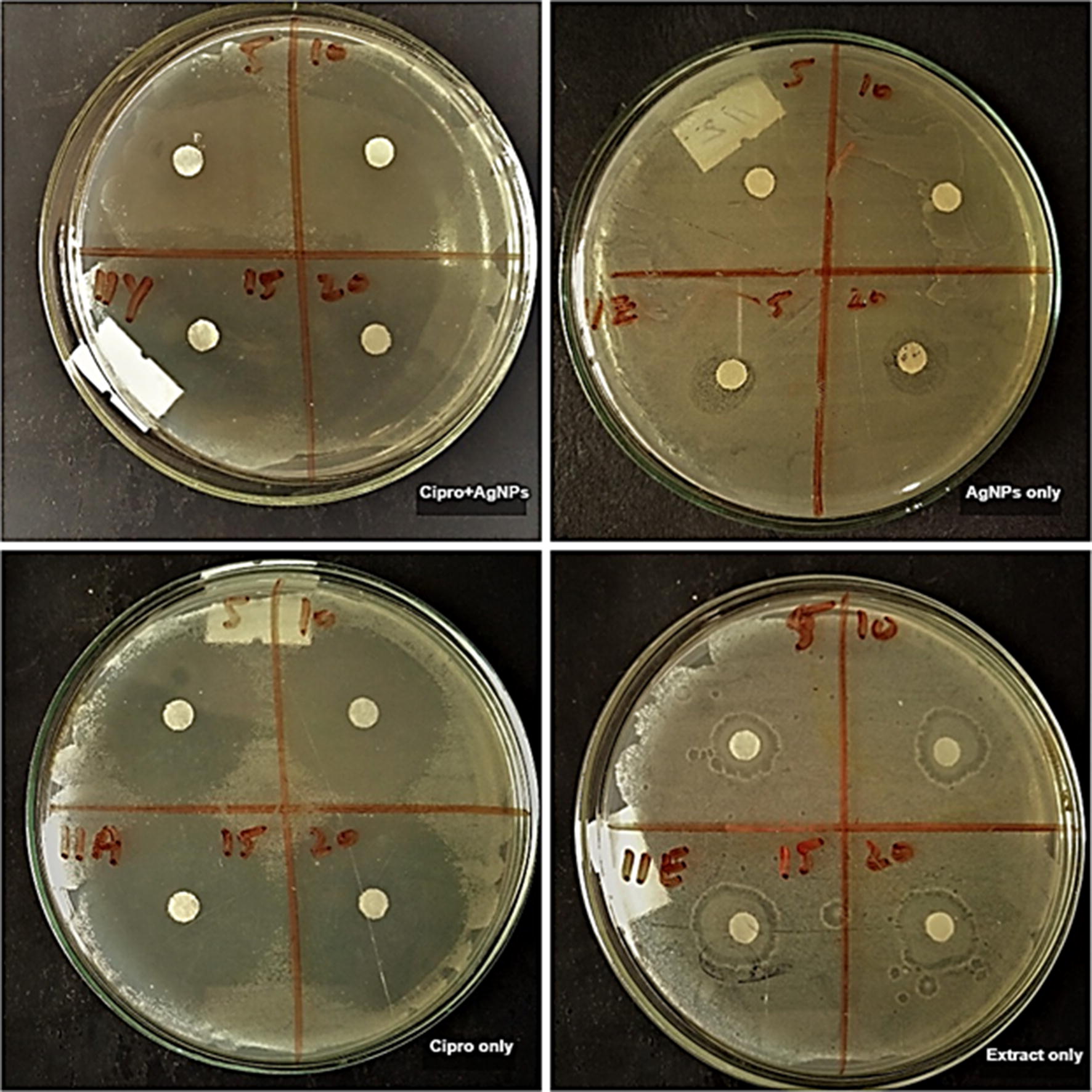
Activity of ciprofloxacin + callus extract mediated AgNPs, callus extract mediated AgNPs, ciprofloxacin only and callus extract only controls against Salmonella typhi

In this study, the antibacterial activity of AgNPs and antibiotics was investigated against pathogenic bacteria *E. coli*,* C. amalonaticus*,* S. sonnei*, and *S. typhi.* The interactive effects of AgNPs with antibiotics was also investigated against these pathogenic strains. Different concentrations (5, 10, 15 and 20 µL) of callus extract mediated AgNPs, ciprofloxacin + callus extract mediated AgNPs, antibiotic alone and callus extract alone was loaded on the sterile disks. Antibiotic alone and callus extract alone were used as a control for the activity. For both types of AgNPs and controls, the activity was performed on separate plates against each strain of bacteria. The zones of inhibitions are recorded in Table 1. For all the four strains no zone of inhibition was observed in the case of callus extract. Callus extract mediated AgNPs showed maximum inhibition zones of 12 mm, 11.5 mm, 12.5 mm and 13 mm in *E. coli*, *C. amalonaticus*,* S. sonnei*, and *S. typhi* respectively. Maximum inhibition zone produced by ciprofloxacin alone were 34 mm, 34 mm, 36.5 mm, and 35.5 mm in *E. coli*,* C. amalonaticus*,* S. sonnei*, and *S. typhi* respectively. Maximum inhibition zone of ciprofloxacin + callus extract mediated AgNPs to *E. coli*,* C. amalonaticus*,* S. sonnei*, and *S. typhi* was 38.5 mm, 35.5 mm, 33 mm, and 35.5 mm, respectively. In the case of all the bacteria except *S. sonnei*, the combination of ciprofloxacin and AgNPs showed an increase in the zones of inhibition as compared to AgNPs and ciprofloxacin alone. The combination of ciprofloxacin and AgNPs produced good results. In the figures, it can be observed that the inhibition zones produced by the association of ciprofloxacin and AgNPs are larger than the inhibition zones of ciprofloxacin alone and AgNPs alone (Figs. 4, 5, 6, 7). The results revealed that the antibiotics associated AgNPs showed better antibacterial activity and produced highest zones as compared to AgNPs and antibiotic alone. From this study, it can be suggested that the AgNPs along with ciprofloxacin might have worked in interaction and resulted in a better antibacterial activity against almost all the tested pathogens. However, the combination of ciprofloxacin and AgNPs was not much effective against *S. sonnei*. The inhibition zones, in this case, were smaller as compared to ciprofloxacin only.

**Table 1:** Average zones of inhibition of different bacteria grown in the presence of callus extract mediated AgNPs, ciprofloxacin + callus extract medicated AgNPs and ciprofloxacin only as control

Tested pathogens	Average zones of inhibition (mm)												
	Callus extract mediated AgNPs (µL)					Ciprofloxacin only (µL) as control				Ciprofloxacin + callus extract mediated AgNPs (µL)			
	5		10	15	20	5	10	15	20	5	10	15	20
*Escherichia coli*	7.5		10.5	12	12	26	29.5	33	34	31.5	35	35	38.5
*Citrobacter amalonaticus*	7		8	9	11.5	23	31	33	34	28.5	30.5	33.5	35.5
*Shigella sonnei*	7		9	9.5	12.5	29	35	35	36.5	26.5	29	30.5	33
*Salmonella typhi*	7		0	11.5	13	29	31.5	34	35.5	29.5	34	35	35.5

## Discussion

It is generally observed that the λ_max_ is between 410 and 40 nm for the AgNPs to have a desired size. However, there is a shift in the surface plasmon resonance peaks toward higher wavelengths in the case of ciprofloxacin + callus extract mediated AgNPs. The reason for this shift has been suggested to be the involvement of ciprofloxacin in the reduction process for the biosynthesis of AgNPs (Shah et al. 2014).

After the formation of callus extract mediated AgNPs, there are some shifts of peaks such as the N–H vibrations from 3440  to 3441 cm^−1^, the N–H or O–H vibrations from 3361 to 3346 cm^−1^ and 3220 cm^−1^ to 3284 cm^−^1, and N–H vibration of amide I from 1626 cm^−1^ to 1637 cm^−1^ (Fig. 2b). The shift of peaks and the disappearance of peaks at 3508 cm^−1^, 3411 cm^−1^, 3253 cm^−1^, 1524 cm^−1^, and 1228 cm^−1^ confirms the involvement of the above-mentioned functional groups in the reduction process. The FTIR spectra of ciprofloxacin showed distinct peaks at 3405 cm^−1^, 1724 cm^−1^, 1628 cm^−1^, 1473 cm^−1^, 1268 cm^−1^ and 1041 cm^−1^ (Fig. 2d). The peaks at 3404 cm^−1^ and 1724 cm^−1^ corresponds to the O–H stretching vibrations and C=O stretching of carboxylic acid, respectively. The peak at 1628 cm^−1^ corresponds to the N–H bending vibration of quinolones. The peak at 1473 cm^−1^ corresponds to the C–O bonding vibrations of the carbonyl group. And similarly, the peak at 1268 cm^−1^ corresponds to the bending vibration of O–H while the peak at 1041 cm^−1^ corresponds to the C–F stretching vibrations. After the formation of ciprofloxacin + callus extract mediated AgNPs, the shifts, and disappearance of some peaks of both ciprofloxacin and callus extracts of *F. indica* were noted (Fig. 2c). In case of callus extract, the N–H vibrations of amide II moved from 3508 cm^−1^ to 3501 cm^−1^ and 3411 cm^−1^ to 3405 cm^−1^. The N–H or O–H bonding vibrations moved from 3361 cm^−1^ to 3337 cm^−1^ and 3220 cm^−1^ to 3298 cm^−1^. The N–H vibrations of amide I moved from 1627 cm^−1^ to 1632 cm^−1^. The peaks at 3440 cm^−1^, 3253 cm^−1^, 1524 cm^−1^ and 1228 cm^−1^ disappeared. In case of Ciprofloxacin, the peak at 3404 cm^−1^ moved to 3405 cm^−1^. The peaks at 1724 cm^−1^, 1473 cm^−1^, 1268 cm^−1^ and 1041 cm^−1^ disappeared. The disappearance of peaks at 3440 cm^−1^, 3253 cm^−1^, 1524 cm^−1^ 1724 cm^−1^, 1473 cm^−1^, 1268 cm^−1^, 1228 cm^−1^, and 1041 cm^−1^ shows the role of the above functional groups of both callus extract and ciprofloxacin in the reduction process and confirms that the reduction occurred. Further, the Bragg peaks representative of silver nanocrystals were also observed. The extra peaks representation of the organic compounds which are there in the plant callus extract. It might be considered that the unassigned peaks are owing to the crystallization of bioorganic phases that occur on the surface of the AgNPs. The current XRD data for 2θ positions provides evidence of the reaction product as silver crystals. Thus, the XRD analysis has shown that the current protocol has resulted in well-defined dimension AgNPs. This also infers that the *F. indica* extracts could be used to effectively synthesize AgNPs (Mehta et al. 2017).

The continuously developing resistance of pathogens is a threat to human life. The antibiotic resistance profiles may lead to the development of multidrug-resistant pathogens and parasites (Tenover 2006). Once an MDR bacteria infects an individual, it is then difficult to cure easily and the person will require broad-spectrum antibiotics for the treatment, which are more toxic, less effective and more expensive (Webb et al. 2005). Thus, the development of potent antimicrobial agents to enhance the bactericidal potential has attracted the interest of the researchers (Lara et al. 2010). Therefore, researchers are trying to use nanoparticles in general and AgNPs in particular to overcome the problem of multidrug-resistant bacteria (Gemmell et al. 2006).

AgNPs possess efficient antibacterial activity (Rai et al. 2012). Previous reports have proposed and explained the mechanism of its antibacterial activity (Nikparast and Saliani 2018). Among the suggested mechanisms, the distribution of the cell membrane of bacteria is highly accepted. It was reported that the AgNPs highly interact with phosphorus and sulfur present in the cell membrane. These elements are utilized by the AgNPs for their antibacterial properties (Guzman et al. 2012). It is also been reported that the attachment of AgNPs to the cell membrane of bacteria results in pits formation on the cell walls, damaging the cell membrane, thus allowing the nanoparticles to pass the periplasm of the bacteria. It has also been reported that the physical and chemical properties of the bacterial cell wall and cell membranes alters due to attachment of AgNPs. This affects the important functions such as osmoregulation, permeability, respiration, and electron transport (Franci et al. 2015; Sondi and Salopek-Sondi 2004).

As the MDRs are developing resistance against classical antibiotics, the use of AgNPs along with antibiotics might be a better alternative and solution for such problematic treatments (Franci et al. 2015). The interaction observed in this study will be of great importance because the use of a small quantity of AgNPs in association with antibiotics can reduce the antibiotic dose required to achieve a similar result. The combined activity of AgNPs and antibiotics can render the resistance developed by pathogenic strains of bacteria. It has been reported that some bonding reactions such as chelation exist between AgNPs and antibiotics that are responsible for the increase in interactive effect (Fayaz et al. 2010). AgNPs and antibiotics kill bacteria through different mechanisms. If the bacterial strain is resistant to any of them, another antibacterial agent would inhibit the growth of that resistant bacterial strain in a different way (Lara et al. 2010). This makes their interaction a more powerful tool against drug-resistant bacteria. It is been reported that AgNPs are selective towards cell membrane, so they act as drug carrier by transporting antibiotics to the cell surface. AgNPs bind to proteins that contain sulfur resulting in an increase in the permeability of cell membrane. Thus, facilitating antibiotic infiltration into the cell (Ghosh et al. 2012). The combination approaches for avoiding antibiotic resistance are getting much attention to be used for administering a low amount of antibiotics. Thus, decreasing the chances of resistance development by pathogens.

In this study, we have demonstrated the interactive role of *F. indica* callus extract and ciprofloxacin for the synthesis of AgNPs using the callus extract of *F. indica*. The addition of ciprofloxacin in very low doses during biosynthesis of AgNPs proved very effective against the bacteria tested. The results presented in this study are novel, thus could emerge as an alternative to the antibiotic only application against different resistant strains. The development of novel combinations can overcome the MDR developed by different bacteria. However, further studies are required to assess the toxicity level and possible side effects that could arise in using the combination of AgNPs and ciprofloxacin as antibacterial agents.

## Data Availability

All the data in the manuscript is available through the open access article policy of the journal.
